# Among the Legislators

**Published:** 1901-03

**Authors:** 


### AMONG THE LEGISLATORS.

Additional legislation in Michigan has been defeated.

Missouri has defeated one measure in the Legislature and is struggling with another.

Indiana has passed a law intended to define what shall constitute the practice of veterinary medicine in that commonwealth.

Montana has accomplished the establishment of an excellent law regulating meat-inspection and milk-inspection and providing for properly qualified inspectors.

